# Dealing with missing data in laboratory test results used as a baseline covariate: results of multi-hospital cohort studies utilizing a database system contributing to MID-NET^®^ in Japan

**DOI:** 10.1186/s12911-023-02345-7

**Published:** 2023-10-30

**Authors:** Maki Komamine, Yoshiaki Fujimura, Masatomo Omiya, Tosiya Sato

**Affiliations:** 1 Department of Biostatistics, Kyoto University School of Public Health, Yoshida-konoecho, Sakyo-ku, Kyoto, 606-8501 Japan; 2 Office of Medical Informatics and Epidemiology, Pharmaceuticals and Medical Devices Agency, Tokyo, Japan; 3 Head Office, Tokushukai Information System Incorporated, Osaka, Japan; 4 Department of Clinical Biostatistics, Graduate School of Medicine, Kyoto University, Kyoto, Japan

**Keywords:** Pharmacoepidemiology, Observational data, Database, Laboratory test item, Missing data

## Abstract

**Background:**

To evaluate missing data methods applied to laboratory test results used for confounding adjustment, utilizing data from 10 MID-NET^®^-collaborative hospitals.

**Methods:**

Using two scenarios, five methods dealing with missing laboratory test results were applied, including three missing data methods (single regression imputation (SRI), multiple imputation (MI), and inverse probability weighted (IPW) method). We compared the point estimates of adjusted hazard ratios (aHRs) and 95% confidence intervals (CIs) between the five methods. Hospital variability in missing data was considered using the hospital-specific approach and overall approach. Confounding adjustment methods were propensity score (PS) weighting, PS matching, and regression adjustment.

**Results:**

In Scenario 1, the risk of diabetes due to second-generation antipsychotics was compared with that due to first-generation antipsychotics. The aHR adjusted by PS weighting using SRI, MI, and IPW by the hospital-specific-approach was 0.61 [95%CI, 0.39–0.96], 0.63 [95%CI, 0.42–0.93], and 0.76 [95%CI, 0.46–1.25], respectively. In Scenario 2, the risk of liver injuries due to rosuvastatin was compared with that due to atorvastatin. Although PS matching largely contributed to differences in aHRs between methods, PS weighting provided no substantial difference in point estimates of aHRs between SRI and MI, similar to Scenario 1. The results of SRI and MI in both scenarios showed no considerable changes, even upon changing the approaches considering hospital variations.

**Conclusions:**

SRI and MI provide similar point estimates of aHR. Two approaches considering hospital variations did not markedly affect the results. Adjustment by PS matching should be used carefully.

**Supplementary Information:**

The online version contains supplementary material available at 10.1186/s12911-023-02345-7.

## Background

In April 2018, the operation of the Medical Information Database Network (MID-NET^®^) began as a national project aimed at utilizing real-world data for drug safety assessments in Japan [1–4]. MID-NET^®^ is a database systems network consisting of 10 collaborative organizations (23 collaborative hospitals) that can analyze data derived from claim data, diagnosis procedure combination data, and electronic medical record (EMR) data at the individual-level [2].

Laboratory test results derived from EMR data have detailed information on clinical symptoms [5] and are expected to be used for confounding adjustments in drug safety assessments. However, the appropriate use of these test results is difficult since a number of data obtained during routine medical care may be missing in datasets for analysis. Therefore, it is essential to appropriately select and apply existing methods to reduce bias due to missing data (hereinafter referred to as “missing data methods”).

Choosing a missing data method requires an understanding of the missing data sources and missing data mechanism [6–8]. Raebel et al., in their study using the US Food and Drug Administration Mini-Sentinel Distributed Database [6], reported that missing data sources of laboratory test results include testing outside of contracted laboratories, patient location where testing was conducted, patient clinical features, etc. In the study of the 10 MID-NET^®^-collaborative hospitals of the Tokushukai Medical Group [9], authors reported that patient background and setting of ordering system of laboratory tests were the main missing data sources. Although their impact was not evaluated because they were unobserved, the measurement policies by physicians and institutions were considered as potential sources as well. Missing data mechanism can be classified as missing completely at random (MCAR), missing at random (MAR), or missing not at random (MNAR) based on the relationship of missing data probability with missing data and observed values [10] (See Table 1).

**Table 1:** Missing data mechanisms and their examples

**Missing data mechanism**	**Description**	**Example**
**Missing completely at random (MCAR)**	The probability that values are missing is unrelated to either the specific missing values that should have been obtained or the set of observed values.	Missing data due to equipment failure.
**Missing at random (MAR)**	The probability that values are missing depends on the set of observed values but is further unrelated to the specific missing values that should have been obtained.	Missing data of blood glucose can be said to be MAR given age, if younger patients are less (or more) likely to have their blood glucose measured than older patients.
**Missing not at random (MNAR)**	The probability that values are missing is related to the specific missing values that should have been obtained, in addition to the ones actually obtained.	If there are data that are unobtained but can influence the missingness of blood glucose, missing data of blood glucose cannot be said to be MAR but said to be MNAR.

In the utilization of laboratory test results contained in MID-NET^®^ to confounding adjustments, it is not clear what impact different missing data methods have on the result. Furthermore, owing to the differences between hospitals in terms of the proportion of patients with missing data (hereinafter, “missing proportion”) and the relationship between missing data and patient background [9], hospital variations regarding missing data should be well-considered.

In this study, the application of missing data methods was carried out to two scenarios of drug safety assessment adjusted by one laboratory test item. We evaluated the impact that missing data methods/approaches to hospital variations had on the interpretation of effect estimates and results.

## Methods

### Study’s scope

We considered a drug safety assessment to estimate an exposure effect using the Cox proportional-hazards model, which is commonly used in cohort designs. Assuming one laboratory test item as a confounder, we applied five missing methods.

### Database and target hospitals

This study used the database system for MID-NET^®^-collaborative organizations of Tokushukai Medical Group (hereinafter, “Tokushukai database”), which has the largest number of MID-NET^®^- collaborative hospitals (10 hospitals; Supplementary Table S1). Supplementary Table S2 demonstrates the data items used for analysis.

### Definition of missing data

As per a previous study, missing data were defined as “data that would be meaningful for the analysis but not available during a specific period before the first prescription date” [9]. The specific period, based on the results of this prior study, was set to 90 days.

### Missing methods

MAR, the reasoning behind which is explained in Missing methods section, was assumed as the missing data mechanism in this study. The following four methods were considered as missing data methods providing unbiased results (hereinafter, “MAR-based methods”) when both the MAR assumption and the correct model specification used in the missing data method (hereinafter collectively referred to as “missing data models”) were correct: single regression imputation (SRI), multiple imputation (MI), inverse probability weighted (IPW) method, and likelihood-based method [11–14]. In the MID-NET^®^ database system, SAS version 9.4 (SAS Institute Inc., Cary, NC, USA) can be used. Since SRI, MI, and IPW methods are implemented in SAS version 9.4, we adopted these MAR-based methods.

**Fig. 1:**
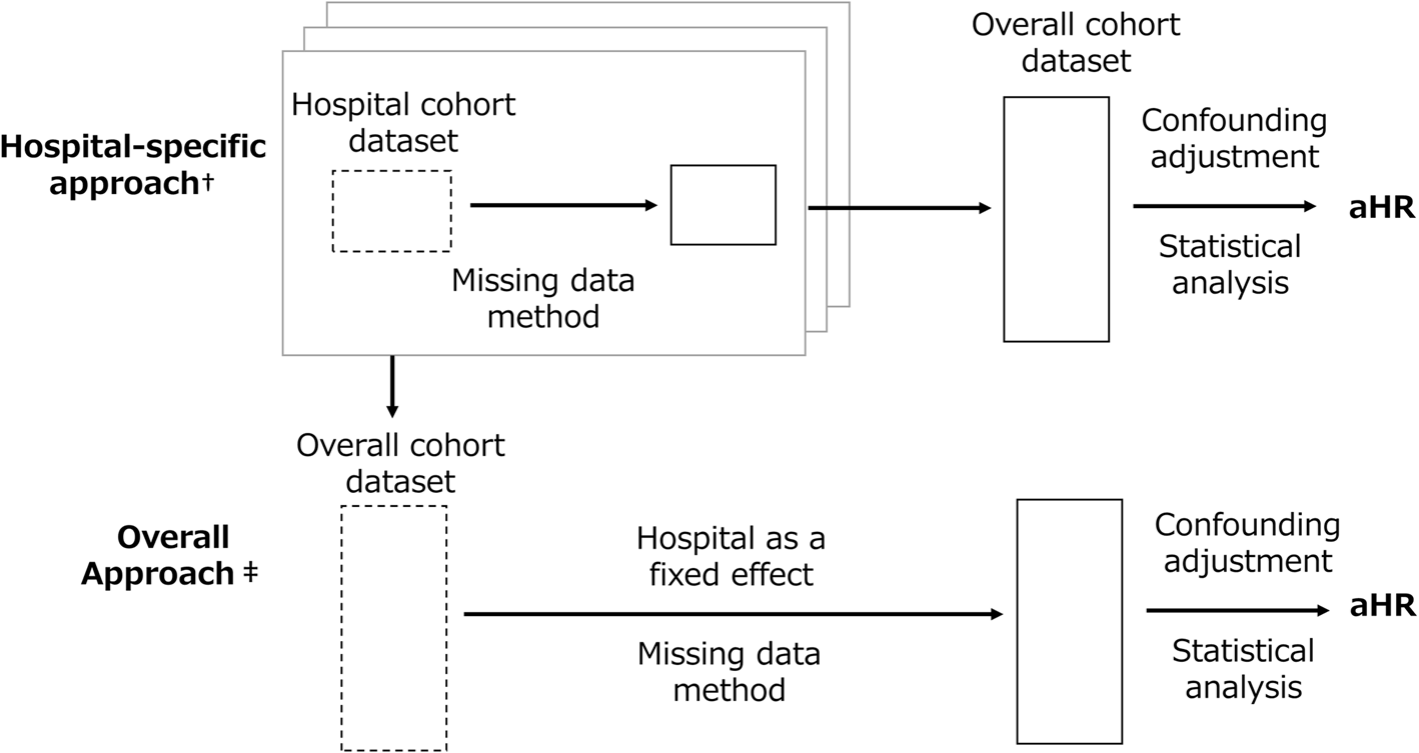
Overview of the two approaches considering hospital variations. Abbreviations: aHR, adjusted hazard ratio. † In the overall approach, the effects of missing data sources at the hospital level, including unobserved missing data sources, can be considered as the hospital effect of the fixed effect, if not all. ‡ In the hospital-specific approach, the interaction of patient background factors with hospitals can be considered

In this study, five missing methods were applied in order to evaluate their impact on the effect estimation of the outcome model. The methods included the above three MAR-based methods, a method excluding a laboratory test item from baseline covariates (Exclusion), and a complete case (CC) method (Table 2). Table 2 provides a brief description of the three MAR-based methods [7, 15–23] and the settings of this study. In the application of MAR-based methods, two approaches were adopted to consider hospital variations in missing data. The hospital-specific-approach implements a missing data model within each hospital cohort. The overall-approach, consequently, implements a missing data model to the overall cohort (combined hospitals cohorts) and uses hospital as a fixed effect covariate (Fig. 1).

**Table 2:** Overview of the five missing methods

**Brief description**	**Limitation/ Concern**	**Setting in this study**
**Excluded a laboratory test item from baseline covariates. (Exclusion)**		• Supplementary evaluation targets.
**Included only patients with laboratory test results (CC method)** **• The method may still yield valid results only if the missing values are not related to the outcome.** **• In the Strengthening the Reporting of Observational Studies in Epidemiology (STROBE) statement **[24]**, it is mentioned that the main differences with the result of the CC method should be discussed when applying MI or IPW method.**		• Supplementary evaluation targets.
**Accounted for missing laboratory test results using SRI** **• The method that assumes an imputation model for the distribution of missing values given the observed values and imputes the missing values with the predicted values obtained.**	• Only one imputation model is created, so it does not take into account the uncertainty of lack of data.	• Imputation model: linear regression model^a^ • The result to be complemented is logarithmic transformation.
**Accounted for missing laboratory test results using MI** **• In this method, after assuming the imputation model, imputation is repeated multiple times (*m*) using random numbers. The parameter estimation of the outcome model is performed for *m* imputed datasets, and the *m* estimates are integrated by Rubin’s rule, etc.** **• The algorithm is complicated, but it can be implemented by available statistical analysis software such as SAS.** **• Uncertainty between complementary models that single regression imputation cannot handle is considered.** **• The imputation model should include not only the predictors of missing values but also the covariates of the outcome model.** **• It is assumed that the distribution of missing values follows a multivariate normal distribution, but there are reports that it is robust even if this assumption does not hold** [15]**.** **• Imputation methods include regression imputation and predicted mean matching.**	• Since it can be implemented by an existing program, it may be implemented without sufficient consideration of the settings of variables to be included in the model.	• Imputation model: linear regression model^a^. • The result to be complemented is logarithmic transformation. • Imputation method: predicted mean matching^b^ • Imputation count: 10 times^c^ • Integration method: Rubin’s rule
**Accounted for missing laboratory test results using the IPW method** **• The method uses an estimation formula weighted by the inverse of the probability of observing the data.** **• A model is assumed for the distribution of the observed probabilities given the observed values, and the complete case is weighted by the inverse of the obtained observed probabilities to estimate the outcome model parameters.** **• A logistic regression model is generally used for the model for the probability of observing the data.**	• Parameter estimation of outcome model becomes unstable in the presence of patients with extremely large weight.	• Model for the probability of observing the data: logistic regression model^a,d^

*Abbreviations*: *CC* Complete case, *IPW* Inverse probability weighted, *MCAR* Missing completely at random, *MI* Multiple imputation, *SRI* Single regression imputation

^a^Included patient background factors (sex, age, year of cohort entry, hospitalization, first visit, emergency care, class number of concomitant medications, complications, and concomitant medication), exposure variables, follow-up period, and event indicators. We referred to the study by White et al. [25] for the follow-up period and event indicator

^b^Predicted mean matching is a method of randomly selecting one observed value close to the predicted value obtained by the imputation model, and it was selected because it can take into account the underestimation of the variance that can occur by regression imputation

^c^Determined by referring to prior research by Raebel et al. [6] that used the Mini-Sentinel Distributed Database

^d^Including outcome information as covariates were referred to the study by Xu Q et al. [26]

### Confounding adjustment and outcome model

For the confounding adjustment methods, we adopted two propensity score (PS) methods (PS weighting and PS matching) as well as an outcome model method with confounding factors as covariates (regression adjustment). The confounding factors, other than the laboratory test item, were patient related factors (see Table 3) and the corresponding hospitals. Since the target population in the scenarios of this study is an exposed population, the standardized mortality ratio weighting (SMRW) [27] was used for PS weighting.

**Table 3:** Details of scenarios 1 and 2

**#**	**Scenario question/ Background**	**Study cohort**	**Target test item with missing**	**Outcome measure**	**Follow-up period**	**Patient-related factors**^**d**^	**Cohort size/ Number (%) of complete cases**
**1**	Whether the risk of diabetes among SGA users is higher than that among FGA users? Glucose metabolic disorders are a known risk of SGA [28, 29]. Therefore, the diabetes risk of SGA is compared to that of FGA.	Inclusion criteria: • Patients initiated with any SGA^a^ or FGA^b^ during the study period (January 01, 2015, to December 31, 2017). • New users of monotherapy for SGA or FGA: Patients with no prescription for the drugs for > 180 days prior to the first prescription of SGA or FGA. Exclusion criteria: • Patients with a diagnosis of dementia (ICD10: F01, F03, G30, G310, etc.) in the 180 days before the first prescription of any SGA or FGA. • Patients with a diagnosis of diabetes (ICD10: E10, E11, E12, E13, E14, O24) in the 180 days before the first prescription of any SGA or FGA.	Blood glucose The blood glucose or HbA1c before prescribing the drug was considered to be the potential confounder. The missing proportions at each hospital cohort were approximately 30–60% for HbA1c and approximately 5–40% for blood glucose. We chose blood glucose because we were interested in the effects of moderate missing data.	Blood glucose ≧200 mg/dL or HbA1c(NGSP)≧6.5% Note: • We convert HbA1c (JDS) to HbA1c (NGSP)	From the date of the first prescription of SGA or FGA to the first date of the following: occurrence of diabetes, end of exposure, end of study period (December 31, 2017), change to a different antipsychotic drug, or addition of a different antipsychotic drug, end of observation period^c^.	Sex, age, year of cohort entry, hospitalization, first visit, emergency care (at the date of the first prescription), class number of concomitant medications, complications^e^, concomitant medication^f^ (180 days prior to the date of the first prescription of any antidiabetic drug)	Overall cohort = 3,430 (SGA users: 1,087, FGA users: 2,343) Complete cases of blood glucose = 2,990 (87.2%)
**2**	Whether users of rosuvastatin have a different risk of hepatic injury than atorvastatin users? Liver damage is a known risk of HMG-CoA reductase inhibitors (statins). Both rosuvastatin and atorvastatin are contraindicated in patients with impaired liver function and have similar levels of alertness for liver damage [30, 31]. Although the results of some observational studies support this [32], atorvastatin (especially at high doses) has been reported to have a higher risk of causing liver damage than other statins [33]. Therefore, the risk of liver damage in rosuvastatin is compared with that in atorvastatin.	Inclusion criteria: • Patients initiated with any rosuvastatin or atorvastatin during the study period (January 01, 2015, to December 31, 2017). • New users of monotherapy for statin as in Scenario 1. Exclusion criteria: Patients with a diagnosis of hepatic injury. (ICD10:B18, K70-K76, K770, K778) within the 180 days before the first prescription of any statin.	ALT, ALP, LDL-chol, TG We selected the above four laboratory tests, thinking that the values of the liver function-related and lipid metabolism-related laboratory tests before prescribing the drug would be potential confounders.	Diagnosis of hepatic injury. (ICD10: K71-K76, K770, K778).	From the date of the first prescription of rosuvastatin or atorvastatin to the first date of the following: occurrence of hepatic injury, end of exposure, end of study period (December 31, 2017), change to another statin, or addition of another statin, end of observation period^c^.	Sex, age, year of cohort entry, hospitalization, first visit, emergency care (at the date of the first prescription), class number of concomitant medication, complications^g^, concomitant medication^h^ (180 days prior to the date of the first prescription of any antidiabetic drug)	Overall cohort = 4,119 (rosuvastatin users: 2,538; atorvastatin users: 1,581) Complete cases of ALT, ALP, LDL-chol, and TG = 3,761 (91.3%), 2,996 (72.7%), 3,176 (77.1%), and 3,306 people (80.3%), respectively.

*Abbreviations*: *ALP* Alkaline phosphatase, *ALT* Alanine transaminase, *FGA* First-generation antipsychotic, *HbA1c* Hemoglobin A1c, *ICD* International Classification of Diseases, *JDS* The Japan Diabetes Society, *LDL-chol* Low-density lipoprotein cholesterol, *NGSP* The National Glycohemoglobin Standardization Program, *SGA* Second-generation antipsychotic, *TG* Triglyceride

^a^Aripiprazole, bronanserin, clozapine, olanzapine, perospirone, quetiapine

^b^Bromperidol, chlorpromazine, clocapramine, fluphenazine, haloperidol, levomepromazine, mosapramine, nemonapride, perphenazine, pimozide, prochlorperazine, propeliciazine, spiperone, sultopride, thimipterone, zotepine

^c^From the perspective of ensuring patient traceability, hospital visits are considered to be consecutive if the interval between visit dates is within a certain range, and we followed these visits to calculate “observation period” per patient

^d^Included in missing data method and confounding adjustment

^e^Hepatitis, liver cirrhosis, chronic pancreatitis, hypertension, hyperlipidemia, hyperthyroidism, Cushing’s syndrome, primary aldosteronism, pancreatic cancer, liver cancer, pheochromocytoma, hemochromatosis, schizophrenia, mood disorder, neurotic disorder, or cancer other than liver and pancreatic

^f^Beta-blockers, thiazide diuretics, antidepressants, corticosteroids, interferon prepared, high-calorie transfusion agents, or immunosuppressants

^g^Chronic kidney disease, heart failure, acute myocardial infarction, hypertension, cerebrovascular diseases, diabetes mellitus, or peripheral vascular disease

^h^Antiepileptic drugs, fibrates, ezetimibe, anti-gout preparations, antithyroid agent, non-steroidal anti-inflammatory drug, antifungal drugs, antituberculosis agents, or therapeutic agents for chronic hepatitis B or C

Then, the point estimate of adjusted hazard ratio (aHR) and 95% confidence interval (CI) were calculated using the Cox proportional-hazard model. In PS matching, stratified Cox proportional-hazard model by matched pairs was used. Robust standard error was used to calculate 95% CI. In the combination of PS methods and MI, aHR estimation was performed for *m* imputed datasets, and *m* estimates were combined (“within approach”) [34] (see Supplementary Fig. S1).

### Scenarios

Based on our previous research [9], we selected the scenarios of two cohort studies that evaluated the relation between drugs and their known risks (see Supplementary Fig. S2). Scenario 1 was the risk of diabetes associated with antipsychotic drug use. In Scenario 1, the blood glucose level before prescription of antipsychotic drugs was the target laboratory test item. Scenario 2 was the risk of hepatic injury associated with HMG-CoA reductase inhibitors (statins) use. In Scenario 2, the target laboratory test item was either the alkaline phosphatase (ALP), alanine transaminase (ALT), low-density lipoprotein cholesterol (LDL-chol), or triglyceride (TG) levels before statins prescription. In Scenario 2, four laboratory test items were included in the baseline covariates of the outcome model, and CC method, SRI, MI, and IPW method were set for each laboratory test item. Table 3 shows the detailed settings of the scenarios, including cohort sizes and the number of complete cases.

### Protocol approval and statistical analysis

Our study protocol was approved by the Kyoto University Graduate School, Faculty of Medicine, and Kyoto University Hospital Ethics Committee in November 2018 (R1793). Statistical analyses were performed using SAS version 9.4 (SAS Institute, Cary, NC, USA).

## Results

Overall cohort sizes and the numbers of complete cases were summarized in Table 3 and Supplementary Figs. S3 and S4. Patient backgrounds including incidence rate are demonstrated in Supplementary Tables S3-S6 (Tables S3 and S5 for overall cohorts and complete cases, and Tables S4 and S6 for hospital cohorts).

### Scenario 1

#### Confounding adjustment by PS weighting

**Fig. 2:**
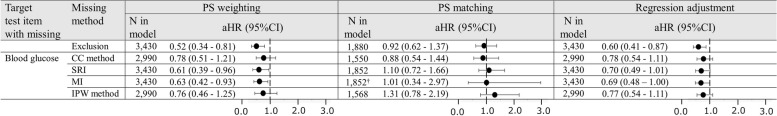
Scenario1 (the risk of diabetes associated with SGA compared to FGA use). Hazard ratios from outcome models with and without baseline blood glucose (SRI, MI, and IPW method were applied by hospital-specific-approach). Abbreviations: aHR, adjusted hazard ratio; CC, complete cases; CI, confidence interval; IPW, inverse probability weighted; MI, multiple imputation; PS, propensity score; SRI, single regression imputation. †The sample size in PS matching with MI gives the mean of 10 matched samples

The aHR of Exclusion was 0.52 (95% CI, 0.34–0.81) (Fig. 2). CC method, SRI, MI, and IPW method contained blood glucose level as a covariate. The aHRs of SRI and MI by the hospital-specific-approach were 0.61 (95% CI, 0.39–0.96) and 0.63 (95% CI, 0.42–0.93), respectively; the point estimates were relatively close, but the width of the 95% CI was slightly narrower in MI. Conversely, the aHRs of the CC and IPW methods by hospital-specific-approach were 0.78 and 0.76, respectively, and thus higher than those of SRI and MI.

**Fig. 3:**
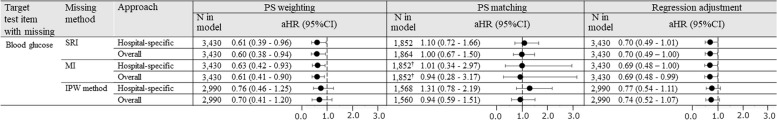
Scenario1 (the risk of diabetes associated with SGA use compared to FGA use). The difference in hazard ratios between approaches considering hospital variations. Abbreviations: aHR, adjusted hazard ratio; CI, confidence interval; IPW, inverse probability weighted; MI, multiple imputation; PS, propensity score; SRI, single regression imputation. †The sample size in PS matching with MI gives the mean of 10 matched samples

The aHRs of SRI, MI, and IPW method by the overall-approach showed no substantial differences compared with the estimates by the hospital-specific-approach (Fig. 3).

#### Confounding adjustment by PS matching

The aHRs of SRI and MI by the hospital-specific-approach were 1.10 (95% CI, 0.72–1.66) and 1.01 (95% CI, 0.34–2.97), respectively. Although there were no substantial differences in point estimates, the width of the 95% CI was larger in MI (Fig. 2). The aHR of the IPW method was 1.31 by the hospital-specific-approach but reduced to 0.94 by the overall-approach (Fig. 3).

#### Regression adjustment

The aHRs of SRI and MI by the hospital-specific-approach were relatively close in terms of the point estimates and the 95% CIs (Fig. 2).

### Scenario 2

#### Confounding adjustment by PS weighting

**Fig. 4:**
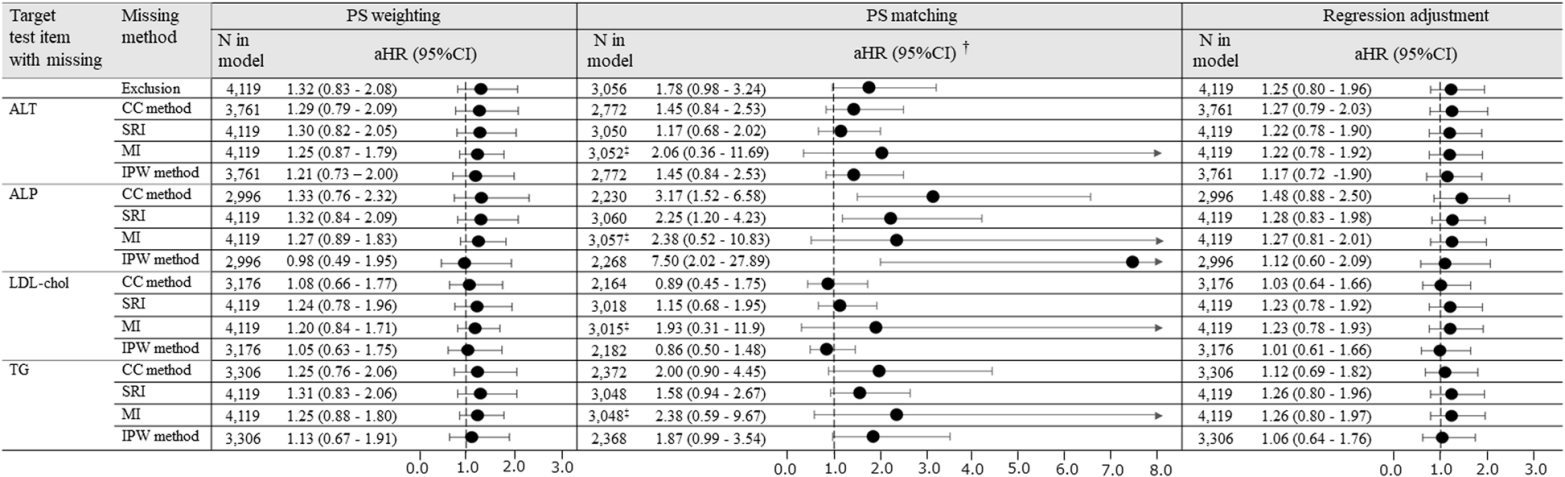
Scenario2 (the risk of hepatic injury associated with rosuvastatin use compared to atorvastatin use). Hazard ratios from outcome models with and without baseline ALT, ALP, LDL-chol, or TG (SRI, MI, and IPW method were applied by hospital-specific-approach). Abbreviations: aHR, adjusted hazard ratio; ALT, alanine transaminase; ALP, alkaline phosphatase; CC, complete cases; CI, confidence interval; IPW, inverse probability weighted; LDL-chol, low-density lipoprotein cholesterol; MI, multiple imputation; TG, triglyceride; PS, propensity score; SRI, single regression imputation. † Not shown in the forest plot if the aHR is 8.0 or more. ‡The sample size in PS matching with MI gives the mean of 10 matched samples

The aHR of Exclusion was 1.32 (95% CI, 0.83–2.08). CC method, SRI, MI, and IPW method included each laboratory test item as a covariate. The aHRs of SRI and MI by hospital-specific-approach varied between 1.20 and 1.32 through all laboratory test items (Fig. 4). While there were no substantial differences in the point estimates of SRI and MI, the width of the 95% CI was slightly narrower in MI; for example, the aHR of SRI and MI for LDL-chol were 1.24 (95% CI, 0.78–1.96) and 1.20 (95% CI, 0.84–1.71), respectively. The aHR of IPW method for ALT was 1.21, whereas those for ALP and LDL-chol were close to 1. The aHRs of the CC method varied depending on the type of laboratory test item. Accordingly, the aHRs for ALT and LDL-chol were 1.29 and 1.08, respectively.

**Fig. 5:**
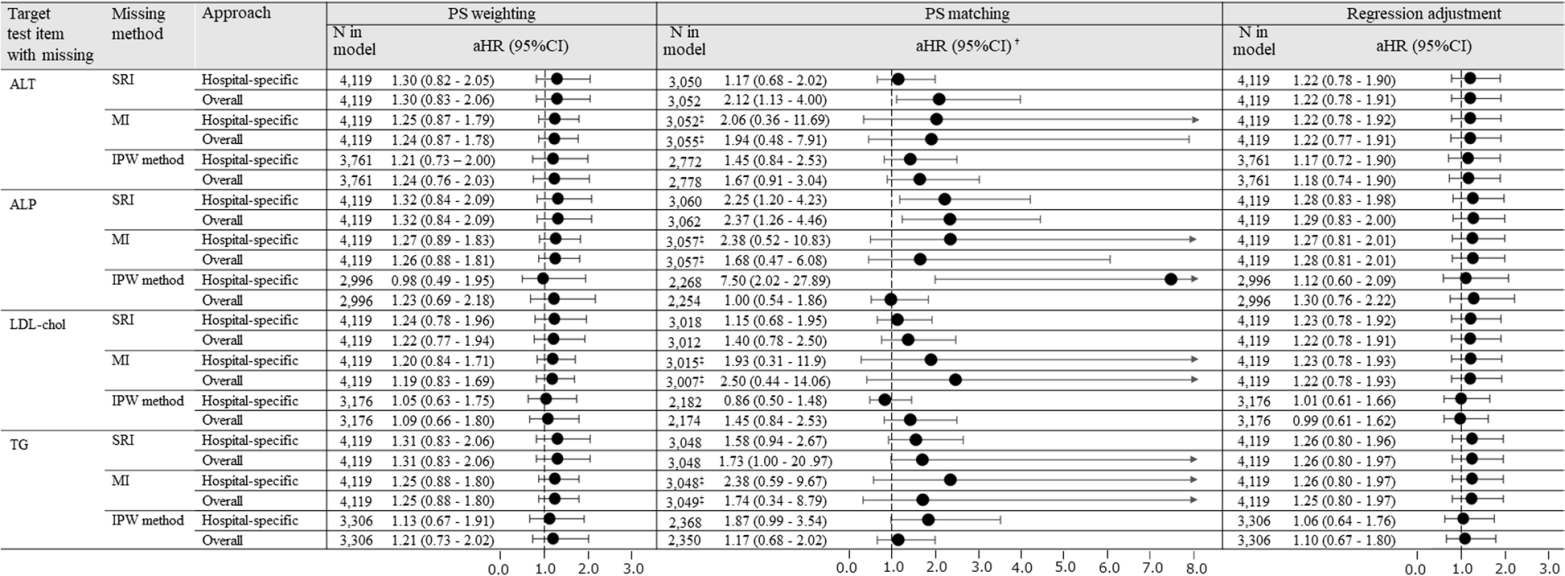
Scenario2 (the risk of hepatic injury associated with rosuvastatin use compared to atorvastatin use). The difference in hazard ratios between approaches considering the hospital variations. Abbreviations: aHR, adjusted hazard ratio; ALT, alanine transaminase; ALP, alkaline phosphatase; CI, confidence interval; IPW, inverse probability weighted; LDL-chol, low-density lipoprotein cholesterol; MI, multiple imputation; TG, triglyceride; PS, propensity score; SRI, single regression imputation. † Not shown in the forest plot if the aHR is 8. or more. ‡The sample size in PS matching with MI gives the mean of 10 matched samples

The aHRs of SRI and MI by the overall-approach ranged between 1.19 and 1.32 through all laboratory test items (Fig. 5). For some laboratory test items, especially ALP, the aHR of MI tended to be higher than those determined by the hospital-specific-approach.

#### Confounding adjustment by PS matching

For all laboratory test items, the differences in aHR between methods were higher and the width of 95% CI was greater than the values obtained with other confounding adjustment methods.

#### Regression adjustment

The aHRs of SRI and MI by the hospital-specific-approach ranged from 1.22 to 1.28 throughout all laboratory test items. For the results in each laboratory test item, both the point estimates and the 95% CIs were relatively close in SRI and MI (Fig. 4). However, the aHR of the IPW method was lower than those of SRI and MI.

## Discussion

We evaluated the impact of five missing methods, approaches to hospital variations, and confounding adjustment methods on the effect estimation in two different scenarios. In Missing methods section of Discusssion and Approaches considering hospital variations sections, we excluded discussions on PS matching in Scenario 2 were excluded and were, instead, included in Confounding adjustment methods section.

### Missing methods

#### SRI versus MI

Although the setting is different, Marshall et al. [35] reported that the bias in SRI and MI, using the same covariates in imputation models, is the same when the missing proportion of a continuous variable is around 25%, and that there was no substantial difference in bias even if the missing proportion increased to 50%. In this study, we had no difference in the point estimates of aHRs between SRI and MI. This might be due to the fact that we used the same covariates in imputation models.

However, for 95% CIs, we found some differences between SRI and MI in confounding adjustment with PS methods (PS weighting of Scenarios 1 and 2 and PS matching of Scenario 1). This will be further explained in Confounding adjustment methods section. Although SRI is generally described as underestimating SE [36], the reason that this study did not show a clear underestimation of SE regardless of the type of confounding adjustment method might be due to the targeted single laboratory test item.

#### IPW method versus imputation methods (SRI and MI)

The degree of difference in the point estimates of aHRs between the IPW method and the imputation methods varied depending on laboratory test items. In particular, there were large differences in Scenarios 1 and 2 adjusted by blood glucose level and ALP, respectively. This might be attributed to the large weights in the IPW method. In the analyses of blood glucose level and ALP, some hospital cohorts had IPWs of 20 or higher. Large weights can contribute to estimation instability. Therefore, it is important to check the stability of the effect estimation using the truncation of large weights [23] as a sensitivity analysis.

#### CC and exclusion methods

In the CC method, large differences in aHRs from those in the MAR-based methods were observed, especially in Scenario 1 adjusted by blood glucose level and in Scenario 2 adjusted by LDL-chol. When the standardized mean differences (SMD) [37] between complete cases and overall cohorts were calculated for patient background factors, factors with SMD of 0.1 or higher existed in Scenarios 1 and 2 (complete cases for LDL-chol) (Supplementary Tables S3 and S5). Such factors were considered to have non-negligible differences in patient backgrounds [38, 39], and the aHR difference between the CC method and the missing data methods was due to the fact that complete cases did not represent overall cohorts.

In the method excluding the laboratory test item in Scenario 1, a relatively large difference in aHR from those in the MAR-based methods occurred. However, in some Scenario 2 cases, the difference was relatively small and no noticeable difference in the analysis for ALT existed. This might be attributed to the fact that confounding by ALT was small because mean ALTs were the same for rosuvastatin and atorvastatin users.

#### Assumption of missing data mechanism

We considered the missing data mechanism in this study was a mixture of MAR and MNAR. Applying the missing data method based on the MNAR assumption requires extensive modeling of the missing data process. In such a case, the MAR-based method may be used as the main analysis method. Then, to evaluate the stability of the main results with the MAR assumption, the pre-planned sensitivity analysis should be considered [40].

When applying the MAR-based methods, the validity of the MAR assumption should be relatively increased by considering as many factors affecting the missing data as possible increases [41, 42]. In this study, all patient background characteristics were included in the missing data model. However, other missing data sources, such as the settings in the ordering system for laboratory tests, the measurement policy of tests, and the preferences of doctors were unobserved and could not be included in the missing data models.

### Approaches considering hospital variations

The results of SRI and MI, and some of those in the IPW method showed no noticeable change in aHR due to different approaches. In the overall approach, hospitals were included in the missing data model as a fixed variable and were expected to capture some of the effects of unobserved missing data sources at the hospital level. In the hospital-specific approach, interactions between patient backgrounds and hospitals can be considered. It is not known whether the hospital effect can be explained by the fixed effect. Additionally, the presence or absence of interaction has not been evaluated, but the results of this study indicate that the difference in approach may not significantly affect the difference in aHRs.

Some changes in aHRs due to the difference in approach in the IPW method may have been caused by the change in the distribution of IPW. In the analysis for ALP in Scenario 2 of the hospital-specific-approach, unlike the overall-approach, some patients with an IPW of 20 or higher existed.

### Confounding adjustment methods

Confounding adjustment methods affected the results in two ways. First, in PS methods (especially PS matching), there was a difference in 95% CIs between SRI and MI. For binary outcomes, Granger et al. [43] reported that coverage of 95% CI was too high in the PS matching compared to the PS weighting using SMRW due to the overestimation of variances. A combination of the PS matching and MI with the “within approach” may have affected the difference in 95% CIs in this study as well.

Second, in the analysis using PS matching, the degree of variation in aHRs and the ranges of 95% CI between missing data methods were large. One of the reasons was attributed to the decrease in the number of patients to be analyzed (Figs. 2 and 3). In Scenario 2, the incidence rate was low (Supplementary Table S5), and the impact of decreasing sample size was large. PS matching should be used carefully, considering the results from this study and its features (e.g., the target population will no longer be an exposed population if all patients in the exposed population do not have matched controls).

In scenario 1, unlike a previous study [44], the increased risk of diabetes associated with SGA use was not observed. In our study, although we did not confirm the frequency of laboratory measurements during the follow-up period, there were differences between groups in the missing proportion before drug prescription (SGA users: 20.6%, FGA users: 9.2%), suggesting that a detection bias may have existed and affected the results. In scenario 2, the point estimates of aHR were around or over 1, consistent with previous studies [32, 33].

When selecting scenarios and laboratory test items, we also considered the feasibility of the number of events; thus, it was not covered in this study, but the following case can also be a typical scenario under which missing laboratory values occur in the MID-NET^®^ based on the knowledge from our previous study [9]; the scenario using laboratory tests with restrictions on the implementation interval based on the health insurance system (e.g., measurement can only be performed once every 3 months) that may be unique to the Japanese medical environment. It is important to Considering the impact and characteristics of missing data is important when planning research.

There are two main limitations to this study. The first is that the scenarios and laboratory test items were limited. Since we used five missing methods, two approaches for considering hospital variations, and three confounder adjustment methods, we had to limit the scenarios and the number of laboratory test items. As more than one laboratory test item can be a confounding factor, further studies focusing on such situations are needed. The second is that we only examined the Tokushukai Database. When applying the missing data method to the entire MID-NET^®^, one must refer to the findings of this study considering the differences from the Tokushukai Database regarding the target population and missing data sources.

## Conclusions

Based on the five main findings of this study (Table 4), we concluded that, although the different missing methods may contribute to differences in parameter estimates of the outcome model, SRI and MI can provide similar point estimates, and two approaches considering hospital variations do not have a major impact on the results. Confounding adjustment by PS matching gave unstable point estimates and wide confidence intervals and should therefore be used carefully. Although we report findings based on a case study and cannot draw generalizable recommendation, our research results may help in the selection of missing data imputation methods and the interpretation of obtained results in the future utilization of MID-NET^®^.

**Table 4:** Main findings in this study

**#**	**Brief description**
**1**	Point estimates for aHR can be similar for SRI and MI, but there may be differences in 95% CI when adjusted with PS methods (especially PS matching)
**2**	In the IPW method and imputation methods, aHR may be similar, but differences can occur.
**3**	In the CC method and the method excluding the laboratory test item, aHR may be similar to that of the MAR-based method, but differences can occur.
**4**	It can be pointed out that the difference in approaches considering hospital variations may not significantly affect the difference in aHR.
**5**	It can be pointed out that the difference in confounding adjustment methods can be different for aHR, especially between PS matching and other methods.

*Abbreviations*: *aHR* Adjusted hazard ratio, *CC* Complete case, *CI* Confidence interval, *IPW* Inverse probability weighted, *MAR* Missing at random, *MI* Multiple imputation, *PS* Propensity score, *SRI* Single regression imputation

### Supplementary Information

**Additional file 1: Supplementary Table S1.** List of the 10 hospitals in the database system for MID-NET^®^ collaborative organizations of Tokushukai Medical Group. **Supplementary Table S2.** Data items used in this study. **Supplementary Table S3.** Scenario 1. Patient backgrounds among the cohort, complete cases, and cases with missing data. **Supplementary Table S4.** Scenario 1. Patient backgrounds by hospital. **Supplementary Table S5.** Scenario 2. Patient backgrounds among the cohort, complete cases, and cases with missing data. **Supplementary Table S6.** Scenario 2. Patient backgrounds by hospital. **Supplementary Figure S1.** Sequence of steps from handling missing data to statistical analysis. **Supplementary Figure S2.** Study scenario selection flowchart. **Supplementary Figure S3.** Scenario 1. Number of patients in the study cohort: risk of diabetes associated with SGA. **Supplementary Figure S4.** Scenario 2. Number of patients in the study cohort: risk of hepatic injury associated with rosuvastatin.

## Data Availability

The datasets generated and/or analyzed during the current study are not publicly available due to the terms of use for MID-NET^®^ to which we adhered when conducting this study; the accessibility of the dataset used for this analysis is restricted to specific authors including the corresponding author in a predetermined secure environment. No outside researchers are allowed to access the dataset. This study used the database system for MID-NET^®^-collaborative organizations of the Tokushukai Medical Group, a part of MID-NET^®^, and not the entire MID-NET^®^. However, we followed the terms of use for MID-NET^®^, as the datasets were included in the entire MID-NET^®^.

## Abbreviations

aHR: Adjusted hazard ratio
ALP: Alkaline phosphatase
ALT: Alanine transaminase
CC: Complete case
CI: Confidence interval
EMR: Electronic medical record
FGA: First-generation antipsychotic
HbA1c: Hemoglobin A1c
ICD: International Classification of Diseases
IPW: Inverse probability weighted
JDS: The Japan Diabetes Society
LDL-chol: Low-density lipoprotein cholesterol
MAR: Missing at random
MCAR: Missing completely at random
MNAR: Missing not at random
MI: Multiple imputation
NGSP: The National Glycohemoglobin Standardization Program
PS: Propensity score
SGA: Second-generation antipsychotic
SMD: Standardized mean differences
SMRW: Standardized mortality ratio weighting
SRI: Single regression imputation
TG: Triglyceride
