# Supplementary material for: Dealing with missing data in laboratory test results used as a baseline covariate: results of multi-hospital cohort studies utilizing a database system contributing to MID-NET® in Japan
Source: BMC Med Inform Decis Mak. 2023 Oct 30;23:242. doi: 10.1186/s12911-023-02345-7 (PMC10617177; doi:10.1186/s12911-023-02345-7)
Supplement: Supplementary file 1 — Additional file 1: Supplementary Table S1. List of the 10 hospitals in the database system for MID-NET® collaborative organizations of Tokushukai Medical Group. Supplementary Table S2. Data items used in this study. Supplementary Table S3. Scenario 1. Patient backgrounds among the cohort, complete cases, and cases with missing data. Supplementary Table S4. Scenario 1. Patient backgrounds by hospital. Supplementary Table S5. Scenario 2. Patient backgrounds among the cohort, complete cases, and cases with missing data. Supplementary Table S6. Scenario 2. Patient backgrounds by hospital. Supplementary Figure S1. Sequence of steps from handling missing data to statistical analysis. Supplementary Figure S2. Study scenario selection flowchart. Supplementary Figure S3. Scenario 1. Number of patients in the study cohort: risk of diabetes associated with SGA. Supplementary Figure S4. Scenario 2. Number of patients in the study cohort: risk of hepatic injury associated with rosuvastatin. [file 12911_2023_2345_MOESM1_ESM.pdf]

Supplementary Information

- File name: Supplement.pdf
- File format: PDF
- Title of data: Supplementary Tables and Figures
- Description of data:

  Supplementary Table S1. List of the 10 hospitals in the database system for MID-NET® collaborative organizations of Tokushukai Medical Group

  Supplementary Table S2. Data items used in this study

  Supplementary Table S3. Scenario 1. Patient backgrounds among the cohort, complete cases, and cases with missing data

  Supplementary Table S4. Scenario 1. Patient backgrounds by hospital

  Supplementary Table S5. Scenario 2. Patient backgrounds among the cohort, complete cases, and cases with missing data

  Supplementary Table S6. Scenario 2. Patient backgrounds by hospital

  Supplementary Figure S1. Sequence of steps from handling missing data to statistical analysis

  Supplementary Figure S2. Study scenario selection flowchart

  Supplementary Figure S3. Scenario 1. Number of patients in the study cohort: risk of diabetes associated with SGA

  Supplementary Figure S4. Scenario 2. Number of patients in the study cohort: risk of hepatic injury associated with rosuvastatin

SUPPLEMENTARY TABLES

Supplementary Table S1. List of the 10 hospitals† in the database system for MID-NET® collaborative organizations of Tokushukai Medical Group

Uji Tokushukai Hospital
Kishiwada Tokushukai Hospital
Sapporo Tokushukai Hospital
Shonan Fujisawa Tokushukai Hospital
Tokyo-Nishi Tokushukai Hospital
Nagoya Tokushukai General Hospital
Nozaki Tokushukai Hospital
Fukuoka Tokushukai Hospital
Matsubara Tokushukai Hospital
Yao Tokushukai General Hospital

MID-NET® is a distributed closed network system in which a database system is built for each collaborative organization. The 10 collaborative organizations include three group hospitals and seven national university hospitals. The data of collaborative hospitals of the group is collected in the database system of the group hospital.

† These are diverse in terms of scale and regionality, but they are all general hospitals with an emergency room, providing core medical care for each region

Supplementary Table S2. Data items used in this study

Data sources	Data items
EMR data	Patient identifiers
	Diagnostic orders data
	Discharge summary data
	Prescription orders/execution data
	Injection orders/execution data
	Laboratory test data
Claim data	Medical procedure data†

† Used to confirm whether it was their first visit and emergency care.

Supplementary Table S3. Scenario 1. Patient backgrounds among the cohort, complete cases, and cases with missing data

	Cohort			CC	Missing
	FGA	SGA	Overall		
	N=2,343	N=1,087	N=3,430	N=2,990	N=440
**Number of events**	131	55	186	179	NA
**Total follow-up periods (person-years)**	90.1	159.5	249.6	138.0	111.6
**Incidence rate(/one person-year)**	1.45	0.34	0.75	1.30	NA
**Sex, male† (%)**	61.2	53.2	58.7	59.8	50.7
**Age†, mean (SD)**	72.5 (13.4)	68.3 (24.4)	71.2 (17.7)	73.6 (14.2)	54.8 (28.0)
**Year of cohort entry†**					
2015	19.3	18.5	19.0	19.1	18.4
2016	41.6	42.6	41.9	42.2	39.5
2017	39.1	38.9	39.1	38.6	42.0
**Hospitalization† (%)**	76.7	69.5	74.4	83.3§	13.9
**Class number of concomitant medications‡, mean (SD)**	0.8 (0.9)	0.6 (0.8)	0.7 (0.8)	0.8 (0.9)	0.3(0.6)
**Complications‡ (%)**					
Hepatitis	4.7	2.6	4.0	4.5	NA
Liver cirrhosis	2.0	1.7	1.9	2.1	NA
Chronic pancreatitis	0.4	NA	0.5	0.5	0
Hypertension	11.8	11.8	11.8	12.8	4.5
Hyperlipidemia	6.5	7.2	6.7	7.4	2.5
Hyperthyroidism	2.7	3.6	3.0	3.2	NA
Cushing’s syndrome	0.7	1.7	1.0	1.2	NA
Primary aldosteronism	NA	0	NA	NA	NA
Pancreatic cancer	3.4	1.2	2.7	3.0	NA
Liver cancer	6.2	2.3	5.0	5.5	NA
Pheochromocytoma	NA	0	NA	NA	NA
Hemochromatosis	NA	0	NA	NA	NA
Schizophrenia	7.3	23.0	12.2	10.0	27.5
Mood disorder	3.6	6.2	4.4	4.2	5.9
Neurotic disorder	5.7	3.9	5.1	5.1	5.5
Cancer other than liver and pancreatic	49.8	25.9	42.2	45.4	20.5
**Concomitant medication‡ (%)**					
Beta-blockers	20.3	19	19.9	22.0	5.2
Thiazide diuretics	3.8	3.8	3.8	4.0	2.5
Antidepressants	6.4	12.9	8.5	7.8	13
Corticosteroids	31.6	15.9	26.6	28.6	13
Interferon prepared	NA	0	NA	NA	NA
High-calorie transfusion agents	7.4	2.2	5.7	6.6	0
Immunosuppressant	0.9	NA	0.8	0.9	NA
**First visit† (%)**	1.1	1.7	1.3	1.1	2.5

**Emergency care† (%)**	27.7	28.0	27.8	31.5	NA

Some categories in which the patient numbers did not exceed 10 were not shown due to privacy.

NA is the case when the number of patients was less than 10.

There was no missing data for sex, age, and year of cohort entry.

Abbreviations: CC, complete cases; FGA, first-generation antipsychotic; SGA, second-generation antipsychotic.

† At the date of the first prescription of any antidiabetic drug. "First visit" and "emergency care" were defined by whether the associated codes were recorded on the same day as the first prescription of the drug of interest.

‡ 180 days prior to the date of the first prescription of any antidiabetic drug.

§ Standardized differences between cohort and CC with greater than 0.1.

Supplementary Table S4. Scenario 1. Patient backgrounds by hospital

	Hospital #									
	1	2	3	4	5	6	7	8	9	10
	N=174	N=121	N=463	N=505	N=388	N=531	N=563	N=208	N=276	N=201
**Sex, male† (%)**	54.0	58.7	64.8	59.2	57.7	58.2	60.0	43.3	56.5	65.2
**Age†, mean (SD)**	70.7 (15.5)	75.9 (11.1)	71.3 (13.9)	70.2 (19.5)	70.1 (17.7)	71.4 (15.4)	74.2 (12.6)	75.1 (17.3)	76.1 (13.6)	52.8 (32.0)
**Year of cohort entry†**										
2015	16.7	19.0	19.7	19.8	17.5	18.5	19.9	25.5	17.8	14.9
2016	35.6	32.2	43.2	46.7	40.7	42.6	43.0	35.1	44.6	38.8
2017	47.7	48.8	37.1	33.5	41.8	39.0	37.1	39.4	37.7	46.3
**Hospitalization† (%)**	66.7	75.2	79.5	72.5	73.7	81.4	66.3	77.4	92.8	51.7
**Class number of concomitant medications‡, mean (SD)**	0.7 (0.8)	0.7 (0.8)	0.9 (0.9)	0.8 (1)	0.7 (0.8)	0.8 (0.8)	0.6 (0.8)	0.6 (0.8)	0.7 (0.8)	0.4 (0.7)
**Exposure (%)**										
FGAs	72.4	74.4	90.5	65.5	54	68.4	73.5	66.8	55.4	48.8
SGAs	27.6	25.6	9.5	34.5	45.9	31.6	26.5	33.2	44.6	51.2
**Baseline result available (%)**										
Blood glucose	86.2	86.8	94.8	86.7	87.9	92.3	84.2	89.4	90.2	58.7
**Complications‡ (%)**										
Hepatitis	NA	NA	5.4	5.1	4.1	3.2	2.1	4.8	5.4	NA
Liver cirrhosis	NA	NA	NA	2.6	3.6	1.9	NA	NA	NA	NA
Chronic pancreatitis	NA	0.0	NA	NA	0.0	NA	NA	NA	NA	NA
Hypertension	11.5	13.2	12.7	11.5	8.0	14.5	12.1	12.5	11.2	9.0
Hyperlipidemia	NA	NA	4.8	4.0	8.0	5.8	12.1	6.3	7.2	6.0
Hyperthyroidism	NA	NA	NA	2.4	2.6	6.4	NA	4.8	5.1	NA
Cushing's syndrome	0.0	0.0	NA	0.0	7.5	NA	NA	0.0	0.0	0.0
Primary aldosteronism	0.0	0.0	0.0	0.0	0.0	0.0	NA	0.0	0.0	0.0
Pancreatic cancer	NA	NA	5.0	3.4	NA	2.3	2.3	NA	NA	NA

Liver cancer	7.5	NA	4.3	6.3	5.9	3.4	5.7	10.6	NA	NA
Pheochromocytoma	0.0	0.0	0.0	0.0	NA	0.0	0.0	0.0	0.0	0.0
Hemochromatosis	0.0	NA	0.0	0.0	0.0	0.0	0.0	0.0	0.0	0.0
Schizophrenia	6.3	24.0	5.8	15.4	8.5	10.9	19.7	11.1	5.4	17.4
Mood disorder	NA	NA	4.8	3.6	7.0	4.3	4.4	8.2	NA	NA
Neurotic disorder	NA	8.3	4.5	4.2	2.8	4.0	9.8	7.7	NA	NA
Cancer other than liver and pancreatic	25.9	42.1	46.4	48.9	34.3	45.2	46.2	43.3	38.8	29.9
**Concomitant medication‡ (%)**										
Beta-blockers	22.4	20.7	26.6	19.8	24.0	19.8	13.0	15.4	25.0	11.4
Thiazide diuretics	NA	NA	3.0	7.1	4.9	2.8	2.1	5.3	4.3	NA
Antidepressants	13.2	8.3	8.9	5.9	15.5	10.0	6.6	7.7	NA	7.0
Corticosteroids	18.4	21.5	32.6	31.1	18.3	29.6	28.8	21.2	29.3	15.9
Interferon prepared	NA	0.0	0.0	0.0	0.0	0.0	0.0	0.0	0.0	0.0
High-calorie transfusion agents	NA	9.1	7.1	9.1	4.6	6.0	3.0	7.7	4.7	NA
Immunosuppressant	0.0	0.0	NA	NA	NA	NA	NA	NA	NA	NA
**First visit† (%)**	NA	NA	NA	NA	NA	NA	1.8	NA	NA	NA
**Emergency care† (%)**	24.7	34.7	34.1	43.4	18.8	25.2	27.0	23.1	17.8	16.9

Some categories in which the patient numbers did not exceed 10 were not shown due to privacy.

NA is the case when the number of patients was less than 10.

There was no missing data for sex, age, and year of cohort entry.

Abbreviations: CC, complete cases; FGA, first-generation antipsychotic; SGA, second-generation antipsychotic.

† At the date of the first prescription of any antidiabetic drug. "First visit" and "emergency care" were defined by whether the associated codes were recorded on the same day as the first prescription of the drug of interest.

‡ 180 days prior to the date of the first prescription of any antidiabetic drug.

Supplementary Table S5. Scenario 2. Patient backgrounds among the cohort, complete cases, and cases with missing data

	Cohort			ALT		ALP		LDL		TG	
				CC	Missing	CC	Missing	CC	Missing	CC	Missing
	**Atorva statin** N=1,581	**Rosuva statin** N=2,538	**Overall** N=4,119	N=3,761	N=358	N=2,996	N=1,123	N=3,176	N=943	N=3,306	N=813
**Number of events (n)**	28	71	99	90	NA	75	24	87	12	83	16
**Total follow-up periods (person-years)**	817.6	1452,7	2,270.3	2,029.0	241.3	1,574.4	695.9	1,974.4	295.9	1,969.3	301.0
**Incidence rate(/one person-year)**	0.03	0.05	0.04	0.04	0.04	0.05	0.03	0.04	0.28	0.04	0.05
**Sex, male† (%)**	52.8	56.6	55.1	56.0	45.8	56.2	52.4	57.6	46.8	57.0	47.6
**Age†, mean (SD)**	70.3 (12.3)	68.5 (12.3)	69.2 (12.3)	69.5 (12.2)	66.0 (12.7)	69.8 (12.2)	67.5 (12.3)	68.5 (12.5)	71.3 (11.5)	68.6 (12.4)	71.4 (11.6)
**Year of cohort entry†(%)**											
2015	19.7	21.2	20.6	19.9	27.7	19.9	22.4	20.5	20.9	20.1	22.5
2016	39.1	39.0	39.0	39.1	38.3	38.6	40.2	39.3	38.2	39.4	37.3
2017	41.2	39.9	40.4	41.0	34.1	41.6	37.3	40.2	40.9	40.4	40.2
**Hospitalization† (%)**	43.5	37.6	39.9	42.6	10.6	47.1§	20.7	32.7§	63.9	35.5	57.7
**Class number of concomitant medications‡, mean (SD)**	0.5 (0.7)	0.5 (0.7)	0.5 (0.7)	0.5 (0.7)	0.4 (0.6)	0.5 (0.7)	0.4 (0.6)	0.4 (0.7)	0.6(0.7)	0.5 (0.7)	0.5 (0.7)
**Complications‡ (%)**											
Chronic kidney disease	2.8	2.9	2.8	3.1	NA	3.5	1.2	3.1	1.8	3.1	1.7
Heart failure	21.9	27.3	25.2	26.8	8.7	27.5	19.1	29.2	11.7	28.4	12.1
Acute myocardial infarction	10.2	13.4	12.1	12.7	5.9	13.8	7.7	13.6	7.3	12.9	9.1
Hypertension	21.3	19.9	20.4	20.9	15.4	22.1	16.1	19.4	24	20.1	22
Cerebrovascular diseases	9.5	12.2	11.2	11.8	4.7	12.4	7.9	12.6	6.3	12.3	6.4
Diabetes mellitus	43.8	46.8	45.7	48.0	20.7	48.0	39.4	50.8	28.3	49.7	29.2

Peripheral vascular disease	NA	0.6	0.6	0.6	0.0	0.6	NA	0.6	NA	0.6	NA
**Concomitant medication‡ (%)**											
Antiepileptic drugs	0.7	0.6	0.6	0.6	NA	0.7	NA	0.7	NA	0.7	NA
Fibrates	2.5	3.7	3.2	3.2	3.9	3.1	3.7	3.7	1.6	3.6	1.6
Ezetimibe	2.5	4.3	3.6	3.7	NA	3.8	3.0	3.6	3.4	3.5	3.8
Anti-gout preparations	15.3	12.6	13.6	13.7	13.1	14.0	12.6	13.5	14	13.8	13.2
Antithyroid Agent	NA	1.0	0.7	0.7	NA	0.7	NA	0.8	NA	0.8	NA
NSAIDs	21.4	22.2	21.9	22.4	16.8	23.3	18.2	19.0	31.4	20.1	29.2
Antifungal drugs	1.4	1.8	1.7	1.8	NA	1.9	0.9	1.6	1.9	1.6	1.7
Antituberculosis agents	NA	NA	NA	NA	NA	NA	NA	NA	NA	NA	NA
Therapeutic agents for chronic hepatitis B or C	NA	NA	NA	NA	NA	NA	NA	NA	NA	NA	NA
**First visit† (%)**	3.7	3.0	3.3	2.8	8.7	2.5	5.3	3.0	4.1	3.0	4.6
**Emergency care†(%)**	14.5	16.1	15.5	16.9	NA	19.3§	5.3	15.3	16.0	15.5	15.3

Some categories in which the patient numbers did not exceed 10 were not shown due to privacy.

NA is the case when the number of patients was less than 10.

There was no missing data for sex, age, and year of cohort entry.

Abbreviations: ALP, alkaline phosphatase; ALT, alanine transaminase CC, complete cases; LDL-chol, low-density lipoprotein cholesterol; NSAID, non-steroidal anti-inflammatory drug; TG, triglyceride.

† At the date of the first prescription of any antidiabetic drug. "First visit" and "emergency care" were defined by whether the associated codes were recorded on the same day as the first prescription of the drug of interest.

‡ 180 days prior to the date of the first prescription of any antidiabetic drug.

§ Standardized differences between cohort and CC with greater than 0.1.

Supplementary Table S6. Scenario 2. Patient backgrounds by hospital

	Hospital #								
	1	3	4	5	6	7	8	9	10
	N=241	N=529	N=631	N=518	N=662	N=661	N=282	N=281	N=314
**Sex, male[†] (%)**	51.5	59.5	53.4	57.5	56.3	55.7	45.4	55.5	54.8
**Age[†], mean (SD)**	68.3 (13.2)	70.0 (10.6)	68.5 (13.0)	67.7 (12.4)	69.0 (12.0)	71.1 (12.2)	67.1 (12.7)	70.6 (12.2)	69.1 (12.5)
**Year of cohort entry[†](%)**									
2015	25.7	16.8	26.1	20.7	22.4	21.2	18.8	12.8	15.3
2016	36.1	37.8	39.6	41.3	34	38.9	39.4	47.3	41.4
2017	38.2	45.4	34.2	38	43.7	39.9	41.8	39.9	43.3
**Hospitalization[‡] (%)**	17.0	43.1	35.3	46.9	44.3	54.0	23.0	30.2	34.1
**Class number of concomitant medications[§], mean (SD)**	0.6 (0.7)	0.5 (0.7)	0.5 (0.7)	0.4 (0.6)	0.5 (0.7)	0.4 (0.6)	0.5 (0.7)	0.5 (0.7)	0.5 (0.7)
**Exposure (%)**									
Atorvastatin	33.2	36.3	32.3	31.1	51.5	38.1	46.5	36.7	37.3
Rosuvastatin	66.8	63.7	67.7	68.9	48.5	61.9	53.5	63.3	62.7
**Baseline result available (%)**									
ALT	87.6	93.6	88.4	95.0	94.3	92.9	89.7	94.0	79.6
ALP	62.7	58.0	65.5	92.1	92.1	70.8	58.9	71.5	64.6
LDL-chol	76.3	75.4	74.2	81.7	81.1	73.7	78.0	80.8	73.6
TG	73.4	90.7	71.2	89.8	80.7	74.9	79.8	86.1	76.1
**Complications[‡] (%)**									
Chronic kidney disease	NA	3.6	NA	6.8	2.3	2.6	3.5	NA	NA
Heart failure	27.0	36.5	22.2	25.5	22.8	18.0	27.0	33.5	21.7
Acute myocardial infarction	17.4	12.3	9.2	14.3	9.5	12.0	14.2	16.0	10.8
Hypertension	18.7	20.0	21.4	17.6	22.2	24.1	12.1	23.5	18.8
Cerebrovascular diseases	12.9	9.3	13.9	12.2	14.0	13.0	NA	6.8	7.3
Diabetes mellitus	49.4	46.7	39.1	45.9	45.3	49.9	53.2	43.4	40.8

Peripheral vascular disease	NA	NA	NA	NA	NA	NA	NA	NA	NA
**Concomitant medication‡ (%)**									
Antiepileptic drugs	NA	NA	NA	NA	NA	NA	NA	NA	NA
Fibrates	4.1	3.4	2.4	NA	2.7	3.0	5.0	4.3	6.7
Ezetimibe	NA	2.3	2.7	8.5	2.3	2.9	NA	5.3	3.2
Anti-gout preparations	14.9	13.8	14.1	9.5	13.0	13.9	14.9	12.8	18.8
Antithyroid Agent	NA	NA	NA	NA	NA	NA	NA	NA	NA
NSAIDs	29.9	25.1	26.0	16.8	22.7	15.7	24.5	23.1	18.2
Antifungal drugs	NA	NA	1.7	NA	1.8	3.0	NA	NA	NA
Antituberculosis agents	NA	NA	NA	NA	NA	NA	NA	NA	NA
Therapeutic agents for chronic hepatitis B or C	NA	NA	NA	NA	NA	NA	NA	NA	NA
**First visit† (%)**	5.0	NA	2.4	2.3	3.8	2.7	7.4	6.8	3.2
**Emergency care†(%)**	6.2	19.1	20.6	12.4	17.8	20.0	7.8	7.5	11.1

Some categories in which the patient numbers did not exceed 10 were not shown due to privacy.

NA is the case when the number of patients was less than 10.

There was no missing data for sex, age, and year of cohort entry.

Abbreviations: ALP, alkaline phosphatase; ALT, alanine transaminase CC, complete cases; LDL-chol, low-density lipoprotein cholesterol; NSAID, non-steroidal anti-inflammatory drug; TG, triglyceride.

†At the date of the first prescription of any antidiabetic drug. "First visit" and "emergency care" were defined by whether the associated codes were recorded on the same day as the first prescription of the drug of interest.

‡ 180 days prior to the date of the first prescription of any antidiabetic drug.

SUPPLEMENTARY FIGURES

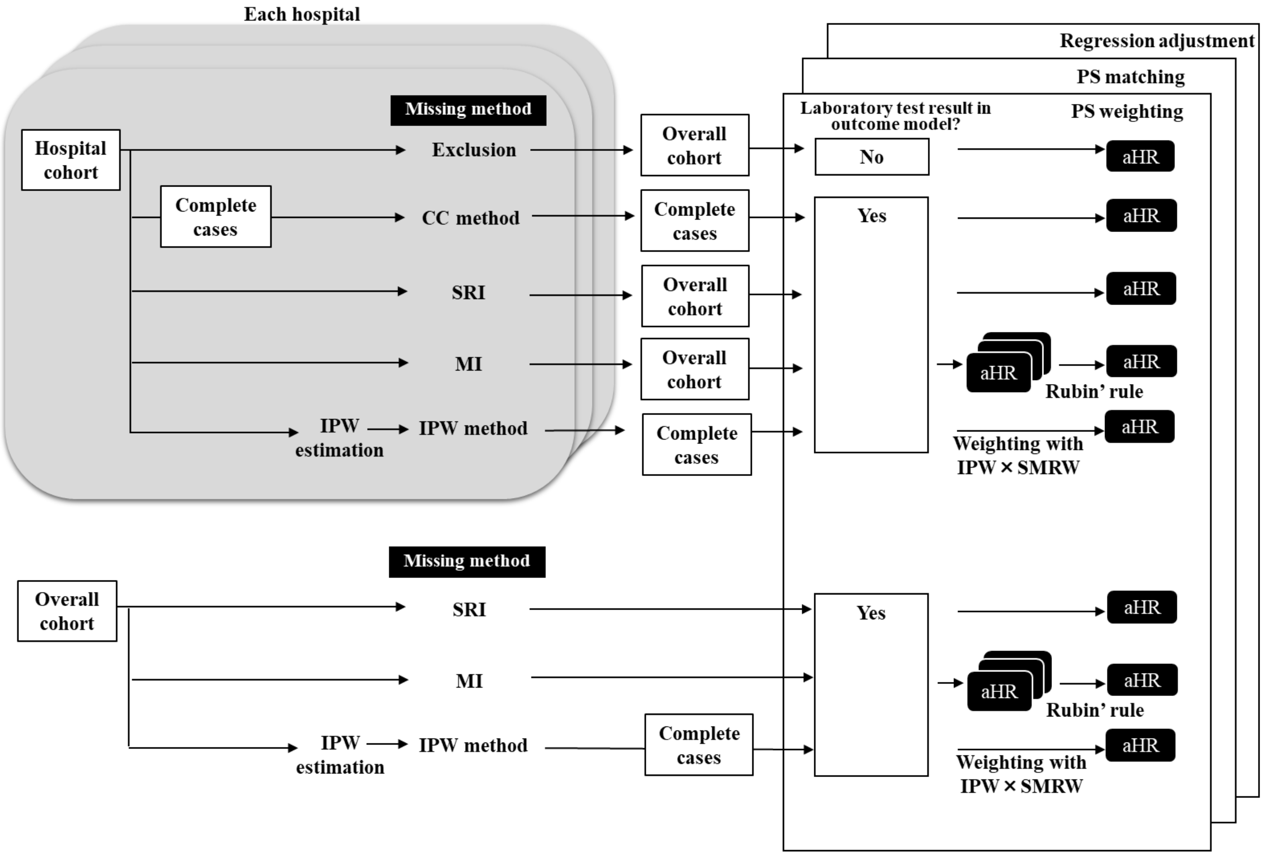

Supplementary Figure S1. Sequence of steps from handling missing data to statistical analysis

PS was estimated by a logistic regression model with confounding factors as covariates. In PS matching, one-to-one greedy matching was performed, and the caliper at the time of matching was 0.2 times the standard deviation of the Logit conversion value of PS. Stratified Cox proportional-hazard model by matched pairs was used as the outcome model in PS matching.

Abbreviations: aHR, adjusted hazard ratio; CC, complete cases; IPW, inverse probability weighted; MI, multiple imputation; PS, propensity score; SMRW, standardized mortality ratio weighting; SRI, single regression imputation.

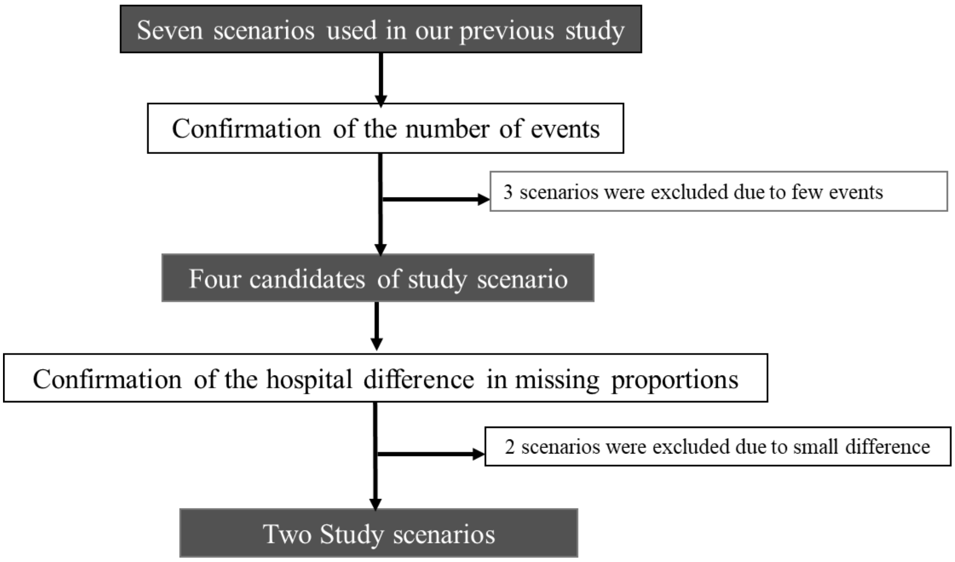

Supplementary Figure S2. Study scenario selection flowchart

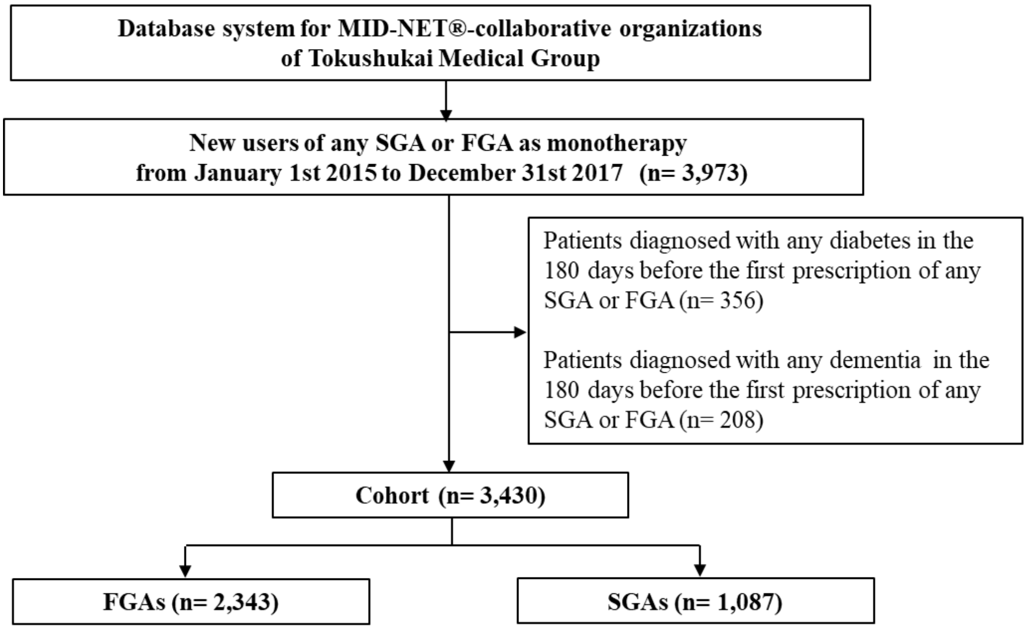

Supplementary Figure S3. Scenario 1. Number of patients in the study cohort: risk of diabetes associated with SGA

Abbreviations: FGA, first-generation antipsychotic; SGA, second-generation antipsychotic.

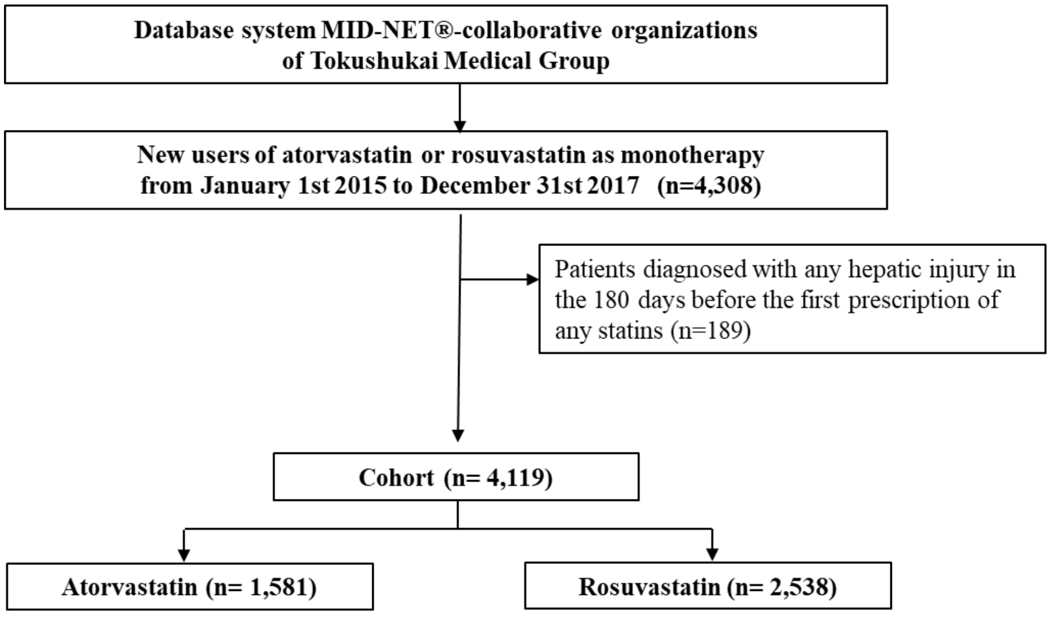

Supplementary Figure S4. Scenario 2. Number of patients in the study cohort: risk of hepatic injury associated with rosuvastatin
